# Unraveling Dowling–Degos Disease: A Rare Skin Disorder

**DOI:** 10.1002/ccr3.70643

**Published:** 2025-07-14

**Authors:** Mahesh Mathur, Neha Thakur, Supriya Paudel, Sambidha Karki, Sandhya Regmi, Nabita Bhattarai

**Affiliations:** ^1^ Department of Dermatology College of Medical Sciences Teaching Hospital Bharatpur Nepal

**Keywords:** Dowling–Degos disease, genodermatosis, histopathology, keratin 5 gene

## Abstract

Dowling–Degos disease (DDD) is a rare genodermatosis characterized by brown to black macules distributed symmetrically in the axilla, groin, elbow, face, neck, and trunk. It is more common in women, usually after puberty. The main pathogenesis behind DDD is a mutation in the *keratin 5* gene. Here, we present a case of 51‐year‐old female presenting as asymptomatic brownish‐black lesions arranged in a reticular pattern involving flexural sites. The clinical and histopathological findings are consistent with DDD. Her mother, brother, son, and daughter also had similar lesions. The patient was counseled about the prognosis and treatment options of the disease.

**Abbreviations**
DDD: Dowling–Degos disease
DSRAD: Dowling–Degos syndrome–related ancestral domain
HPE: histopathological examination
KRT 5: *keratin 5 gene*
POFUT1: protein O fucosyltransferase 1
POGLUT1: protein O‐glucosyltransferase 1
PSENEN: presenilin enhancer protein 2 gene

**Summary**

- Dowling–Degos disease (DDD) is an uncommon autosomal dominant disorder characterized by reticulate pigmentation.
- It is important to diagnose the disease as it mimics many other cutaneous conditions.
- Typical histopathology is pathognomic and aids in the diagnosis of this rare entity.

## 1 Introduction

Dowling–Degos disease (DDD) is a rare, progressive, autosomal dominant disorder characterized by symmetrical, pigmented macules involving the axillae, groins, face, neck, arms, and trunk as well as scattered comedo‐like lesions and pitted acneiform scars [1]. Its prevalence is not well documented in the literature. DDD was described initially by Dowling and Freudenthal in 1938 [2]. Typically, the onset of DDD is after puberty and commonly occurs in the third to fourth decade of life [3]. Women are more commonly affected than men [2]. Histopathological examination (HPE) of skin lesions is diagnostic. We hereby report a case of DDD considering the rarity of this skin condition.

## 2 Case Presentation

**FIGURE 1:**
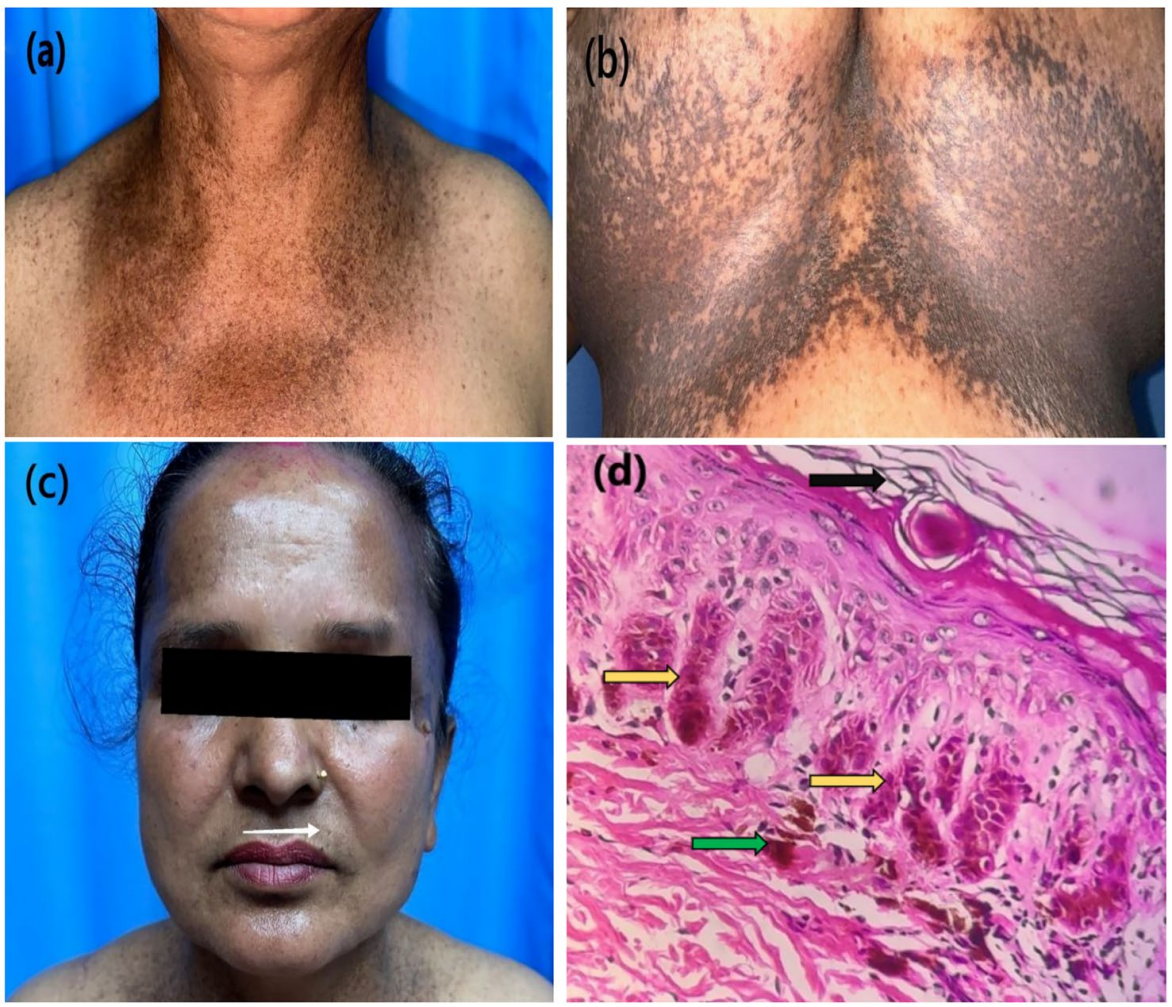
(a) Multiple reticular pigmentations over neck and chest. (b) Over inframammary region. (c) Few pitted scars above the upper lip in left side (white arrow). (d) Histopathology: Hematoxylin and eosin staining (40×) with hyperkeratosis (shown by black arrow), “antler‐like” pattern (shown by yellow arrow) and basal hyperpigmentation (shown by green arrow).

**FIGURE 2:**
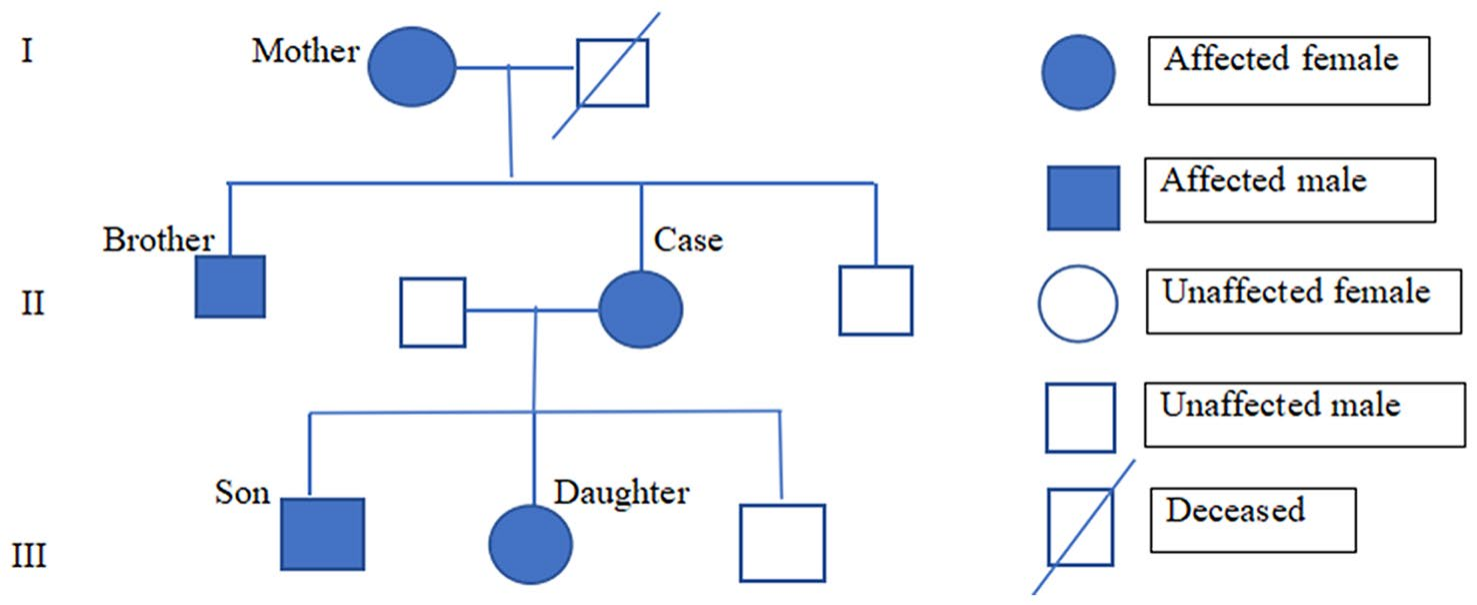
Pedigree of the family with Dowling–Degos disease.

A 51‐year‐old female presented with multiple asymptomatic reticular hyperpigmented macules, which appeared first on the flexor aspect of the elbow and progressed to involve other flexural parts of the body (Figure 1a,b). Skin lesions were present around the age of 25, and the pigmentation has worsened over the past 4 years. There is a history of similar lesions in her mother, brother, son, and daughter (Figure 2 pedigree chart). On examination, there were multiple brown to black colored macules of 2–4 mm in diameter arranged in a reticular pattern distributed symmetrically over the face, chest, inframammary region, axilla, elbow, and groin. Few pitted scars (Figure 1c) were present in the perioral regions.

## 3 Methods

Routine laboratory investigations were within normal limits. HPE of skin biopsy from right inframammary region showed hyperkeratosis, orthokeratosis, focal parakeratosis, and thinning of suprapapillary epidermis. The epithelial strands extend into the superficial dermis from the epidermis and hair follicles resulting in an “antler‐like” pattern (Figure 1d). Basal hyperpigmentation and mild perivascular lymphocytic infiltration in dermis were also noted.

## 4 Results

On the basis of clinical and histological findings, the diagnosis of DDD was made. The patient was counseled about the prognosis of the disease and treatment options.

## 5 Discussion

DDD is a rare autosomal dominant genodermatosis but can also occur sporadically. DDD is clinically characterized by asymptomatic, progressive, reticulate hyperpigmented macules with a flexural distribution. Other cutaneous findings include comedo‐like hyperkeratotic follicular papules on the neck and perioral pitted acneiform scars [4]. In our case, classical reticulated pigmentations were present in flexures; however, comedo‐like papules were not seen.

Loss‐of‐functional mutation in the *keratin 5 (KRT5)* gene on chromosome 12q13 is reported. Mutation of this gene results in disruption in melanosome transfer and trafficking, disrupting the normal skin pigmentation and structure [5]. Other genes involved in pathogenesis are *POGLUT1, POFUT, PSENEN, and DSRAD* gene in chromosome 17p13.3 and chromosome 1q21 [1, 5]. Genetic studies are not carried out in our case due to affordability issues.

Epidermal cysts, multiple keratoacanthomas, squamous cell carcinoma, abscess, hidradenitis suppurativa, seborrheic keratosis, and pilonidal cysts are associated with DDD [2]. These disorders are not present in our case. Histopathology of the skin biopsy in classical DDD reveals thinning of the epidermis, elongated, thinned, and branched (antler‐like) rete ridges with increased melanin pigmentation at their tips, but no increase in the melanocyte number [6]. Histopathology of the skin biopsy of our patient also revealed these classical findings.

Variety of hyperpigmentation disorders appearing in flexural areas that should be differentiated from DDD are Galli–Galli disease, reticulate acropigmentation of Kitamura, Haber syndrome, dyschromatosis symmetrica hereditaria, dyschromatosis universalis hereditaria, neurofibromatosis type 1, and acanthosis nigricans. Details regarding these differential diagnoses are described in Table 1 [7, 8, 9]. This case report highlights the importance of recognizing the clinical and histopathological features of DDD for accurate diagnosis and differentiation from other similar conditions.

**TABLE 1:** Differential diagnosis of Dowling–Degos disease.

Differential diagnosis	Differentiating features
Galli‐Galli disease	Reticulated hyperpigmentation in flexures but supra basal dyskeratotic acantholysis in HPE
Reticulate acropigmentation of Kitamura	Childhood onset, atrophic hyperpigmented papules coalescing into a reticulated pattern in acral areas
Haber syndrome	Photosensitive rosacea‐like facial eruptions initially followed by keratotic papules, comedo‐like lesions, pitted scars, reticulate hyperpigmentation on trunk, proximal extremities and axillae
Dyschromatosis symmetrica hereditaria	Pinpoint, hypo and hyperpigmented macules on dorsal aspects of the hand and feet
Dyschromatosis universalis hereditaria	Pigmented flecks and spots on body parts other than flexures
Neurofibromatosis type 1	Axillary and inguinal freckling with multiple neurofibromas and other cutaneous manifestations
Acanthosis nigricans	Velvety plaques, less elongation of rete ridges and no follicular involvement in HPE

There is no definitive cure for DDD. Various treatment options have been tried in recent years, but none of them have shown satisfactory results. Treatment options include depigmenting agents such as hydroquinone, retinoids, and laser therapies such as fractional erbium YAG laser. Despite these options, effective and long‐term solutions remain difficult to achieve [10].

## 6 Conclusions

DDD is an uncommon and non‐life‐threatening genetic disorder. However, discoloration associated with DDD can lead to psychological distress. Continued research and documentation of such cases may lead to better treatment options in the future.

## Author Contributions

**Mahesh Mathur:** conceptualization, formal analysis, resources, supervision, validation, visualization, writing – original draft. **Neha Thakur:** conceptualization, formal analysis, resources, supervision, validation, visualization, writing – original draft. **Supriya Paudel:** formal analysis, resources, supervision, visualization, writing – original draft, writing – review and editing. **Sandhya Regmi:** data curation, investigation, resources, visualization, writing – review and editing. **Sambidha Karki:** data curation, investigation, resources, visualization, writing – review and editing. **Nabita Bhattarai:** conceptualization, formal analysis, resources, supervision, validation, visualization, writing – original draft.

## Ethics Statement

Reviewed and approved by Institutional review board College of medical sciences (IRBCOMS). The patients in this manuscript have given written informed consent to the publication of their case details.

## Consent

The authors obtained written consent from the patient for use of photographs and medical information to be published online and with the understanding that this information may be publicly available and discoverable via search engines. Patient consent forms are *not* provided to the journal but are retained by the authors.

## Conflicts of Interest

The authors declare no conflicts of interest.

## Data Availability

The data that support the findings of this study are available from the corresponding author upon reasonable request.
